# IL-13 from intraepithelial lymphocytes regulates tissue homeostasis and protects against carcinogenesis in the skin

**DOI:** 10.1038/ncomms12080

**Published:** 2016-06-30

**Authors:** Tim Dalessandri, Greg Crawford, Mark Hayes, Rocio Castro Seoane, Jessica Strid

**Affiliations:** 1 Division of Immunology and Inflammation, Department of Medicine, Imperial College London, London W12 0NN, UK

## Abstract

The skin is under constant renewal and exposure to environmental challenges. How homeostasis is maintained alongside protective mechanisms against damage is unclear. Among the basal epithelial cells (ECs) is a population of resident intraepithelial lymphocytes (IELs) that provide host-protective immune surveillance. Here we show that IELs cross-communicate with ECs via the production of IL-13. Skin ECs are activated by IEL-derived IL-13, enabling a canonical EC stress response. In the absence of IL-13, or canonical IEL, the skin has decreased ability to repair its barrier and increased susceptibility to cutaneous carcinogenesis. IL-13 controls the rate of EC movement through the epidermis, which might explain the importance of IL-13 for epidermal integrity and its suppressive effect on skin carcinogenesis. These findings show that IL-13 acts as a molecular bridge between IELs and ECs, and reveal a critical host-defensive role for type-2 immunity in regulating EC tissue homeostasis and carcinogenesis.

## Abstract (web summary)

Epidermal intraepithelial lymphocytes (IEL) produce IL-13, but the physiological role of this cytokine production is not clear. Here the authors show that IEL-production of IL-13 is a vital lymphoid stress surveillance mechanism driving crosstalk with epithelial cells to maintain tissue homeostasis and inhibit chemical carcinogenesis in mice.

## Graphical abstract

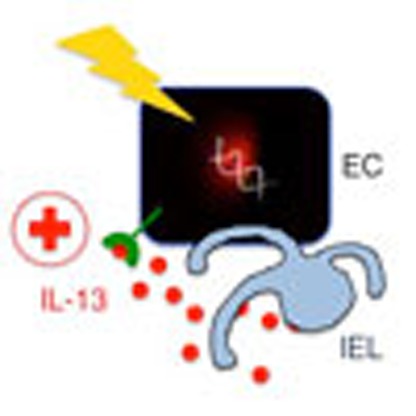

Immune surveillance refers to the capacity of the immune system to sense cellular dysregulation and respond to restore homeostasis. This continued ‘quality control' mechanism has most commonly been studied in relation to cancer^1^,^2^,^3^,^4^. However, the concept of immune surveillance can also be more broadly applied to non-malignant pathologies and homeostatic tissue control^5^.

The epithelial barriers of our body surface tissues are continually exposed to environmental challenges and must respond appropriately to maintain tissue integrity. Epithelial cells (ECs), which line body surface tissues, are dynamic and versatile cells, and evidence indicates that they are important drivers of immune surveillance^6^. In close association with ECs are the specialized tissue-resident T cells known as intraepithelial lymphocytes (IELs). IELs constitute a large, but somewhat enigmatic, population of T lymphocytes. They carry recombination activating gene (RAG)-dependent rearranged T-cell receptors (TCRs), yet have limited TCR diversity and are mainly major histocompatibility complex non-restricted cells. They express innate receptors that enable reaction to stress antigens with rapid ‘innate-like' response kinetics^7^. The murine skin contains a unique subset of γδ TCR^+^ IELs, called dendritic epidermal T cells, that exclusively carry a Vγ5Vδ1 TCR and constitutively express stress-sensing receptors such as NKG2D. NKG2D ligands are induced on stressed ECs, are expressed by most epithelial tumours and the NKG2D pathway is strongly associated with anti-tumour responses in both humans and mice^8^. We have previously shown that skin IELs directly recognize and respond to alterations in autologous stress antigens on local ECs (keratinocytes (KCs)), including ligands for the NKG2D receptor^9^. This rapid afferent sensing of stressed ECs probably has a crucial role in the early detection of pre-malignant cells and has been termed ‘lymphoid stress surveillance' (LSS)^10^,^11^. Indeed, the absence of canonical skin IELs confers a significant increase in the susceptibility to skin carcinogenesis^9^. Curiously, when tumour-protective skin-resident IELs are activated by stressed ECs in the LSS response, a dominant Th2-biased downstream response is triggered with large amounts of interleukin (IL)-13 and IgE being produced. This stress-induced type-2 immune response is dependent on canonical IEL recognition of stressed ECs via the NKG2D pathway^12^. Although Th2 immunity has traditionally been thought to impair host tumour eradication, the surprising association between a stress sensor such as NKG2D and induction of type-2 immunity necessitates investigation into the role of early type-2 immunity in cancer immune surveillance.

Stressed ECs promptly release many cytokines; among the most robustly expressed are IL-25, IL-33 and thymic stromal lymphopoietin (TSLP), all of which can drive type-2 immune responses. This propensity of damaged epithelial tissues to induce type-2 immunity may underlie the high frequency of allergic and atopic disease at the skin and mucosal surfaces. However, despite the intense interest in this area, the cellular and molecular basis of how type-2 immunity is linked with EC dysregulation and barrier disruption is not fully understood, nor is the functional role of this type of immunity for EC homeostasis or immune surveillance fully elucidated. The relationship between allergic disease and cancer has been long-debated, but the biological nature of this association is unclear. Overviews of the epidemiology literature show both potent inverse and positive associations. This divergence highlights the complexity of the underlying interactions as well as reflects the heterogeneity of these diseases. Intriguingly, inverse associations are more common for tissues that interface with the external environment, such as the skin^13^,^14^. Nonetheless, molecular mechanism(s) for how atopy may translate into a distinct functional advantage against EC carcinogenesis have not been described.

Here we investigate the importance of IEL–EC cross-talk and type-2 immunity in skin homeostasis. We show that an important part of LSS is rapid IL-13 production by IELs, and that this provides a molecular bridge between the IELs and ECs. IEL-derived IL-13 has prominent effects on skin ECs, enabling a canonical EC response to stress with the release of TSLP and IL-33, and accelerating the rate of EC movement through the epidermis. Mice lacking IL-13, or canonical IEL, have evidence of stress in the skin, with enhanced transepidermal water loss (TEWL) and defects in the restoration of skin barrier integrity after insult. In addition, mice lacking IL-13 are more susceptible to cutaneous carcinogenesis. Hence, LSS-induced type-2 immunity is part of an early ‘allergic' host defence mechanism regulating tissue homeostasis and protecting against carcinogenesis.

## Results

### Skin IELs are potent producers of IL-13

**Figure 1:**
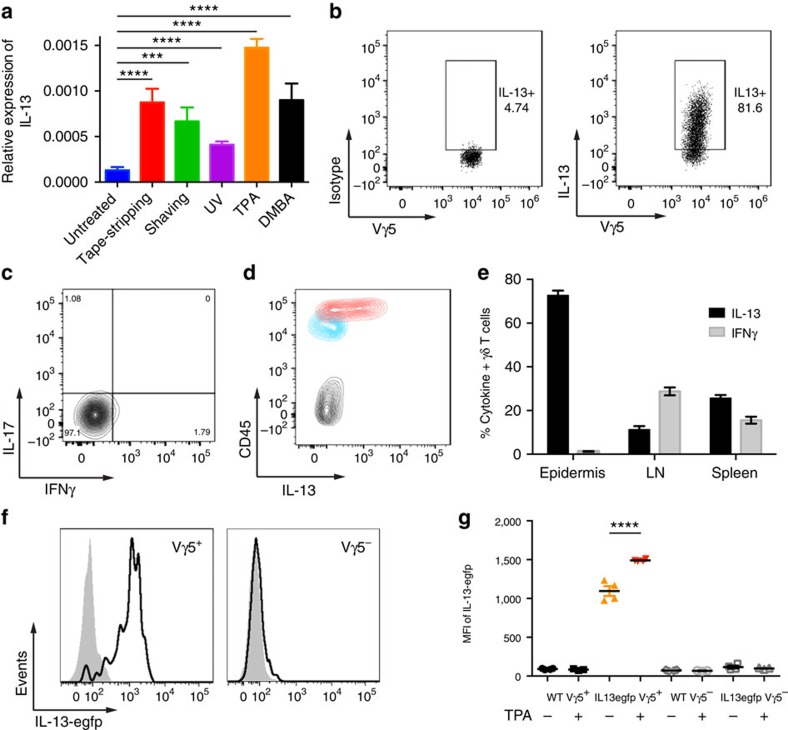
Skin IELs are potent producers of IL-13. (**a**) Quantitative RT–PCR analysis of IL-13 mRNA in the epidermis isolated 4 h after the indicated topical stress. Data are expressed as mean±1 s.e.m. relative to the control gene cyclophylin (*n*=3–6 per condition). (**b**–**d**) Intracellular protein staining in Vγ5^+^ skin IELs following 4 h *ex vivo* stimulation with PMA/iono of (**b**–**d**) IL-13 and (**c**) IFN-γ and IL-17. In **d**, CD45^hi^ Vγ5^+^ skin IELs (red) are shown with CD45^mid^ Langerin^+^ LCs (blue) and CD45^−^ KCs (black). Representative FACS plots shown. (**e**) γδ T cells isolated from the epidermis, LN or spleen compared for intracellular IL-13 and INFγ production following 4 h *ex vivo* stimulation with PMA/Iono (*n*=3). (**a**–**e**) data from WT FVB mice. (**f**) Vγ5^+^ and Vγ5^−^ TCRγδ^+^ T cells isolated from naive skin of BALB/c mice (grey) and IL-13egfp reporter mice (black lines) and (**g**) 24 h after topical exposure to TPA. (**f**) Shows histograms of the gfp^+^ γδ T cells and (**g**) the MFI of IL-13egfp before and after TPA challenge (*n*=3 and 4). Statistical significance of difference was determined using Student's *t*-test for unpaired data with ***P<0.001 and ****P<0.0001.

The importance of tissue-specific IELs for host protection is evident by the marked increase in skin tumour susceptibility of mice lacking only Vγ5Vδ1^+^ skin IELs^9^. The tumour protection conferred by γδ T cells has primarily been attributed to their cytotoxicity and them being an early source of interferon-γ (IFN-γ) (refs 15, 16). However, we found that, when skin IELs were activated *in situ* by a variety of environmental stressors, their principal stress response was to produce type-2 cytokines, primarily IL-13 (Fig. 1). In the resting skin, IELs are morphologically very dendritic and use these dendrites to form contact points with the ECs^17^ (Supplementary Fig. 1). Upon a mild topical physical insult, or skin exposure to chemicals such as 12-*O*-tetradecanoylphorbol-13-acetate (TPA) or the carcinogen 7,12-dimethylbenz[a]anthracene (DMBA), IELs retracted their dendrites and rounded up their cell bodies (Supplementary Fig. 1), which parallels with IEL activation^9^. At the same time, IL-13 was upregulated in the epidermal tissue. This was a conserved response to stress and was seen both after exposure to physical stressors, such as tape-stripping and shaving, or following exposure to ultraviolet irradiation (UV) and chemicals such as TPA or DMBA (Fig. 1a)—and even after transgenic upregulation of NKG2D ligands on skin ECs^12^. The vast majority of freshly isolated skin IELs stimulated *ex vivo* also produced potent amounts of IL-13 protein within just a few hours, while they did not produce IFN-γ or IL-17 (Fig. 1b,c). Importantly, the IELs were the only cells in the epidermis to produce IL-13 (Fig. 1d). This IL-13 signature was unique to the Vγ5Vδ1^+^ skin IELs and not a universal γδ T-cell phenomenon, as systemic γδ T cells isolated from the skin draining lymph node or spleen did not show this IL-13 bias (Fig. 1e; supplementary Fig. 2). Using IL-13-egfp reporter mice, we found that Vγ5Vδ1^+^ epidermal IELs were in fact positive for IL-13 already at steady state in the resting skin, and that IL-13 expression was further upregulated following *in vivo* topical exposure to TPA (Fig. 1f,g) and other environmental challenges (Supplementary Fig. 3). In contrast, Vγ5^−^ dermal γδ T cells were negative for IL-13-egfp (Fig. 1f,g). Hence, the epidermal IELs constitutively produce IL-13 at steady state, show a distinctive and conserved IL-13 signature when responding to environmental stress and are the only cells in the epidermis to produce IL-13.

### IEL-derived IL-13 activates skin ECs

**Figure 2:**
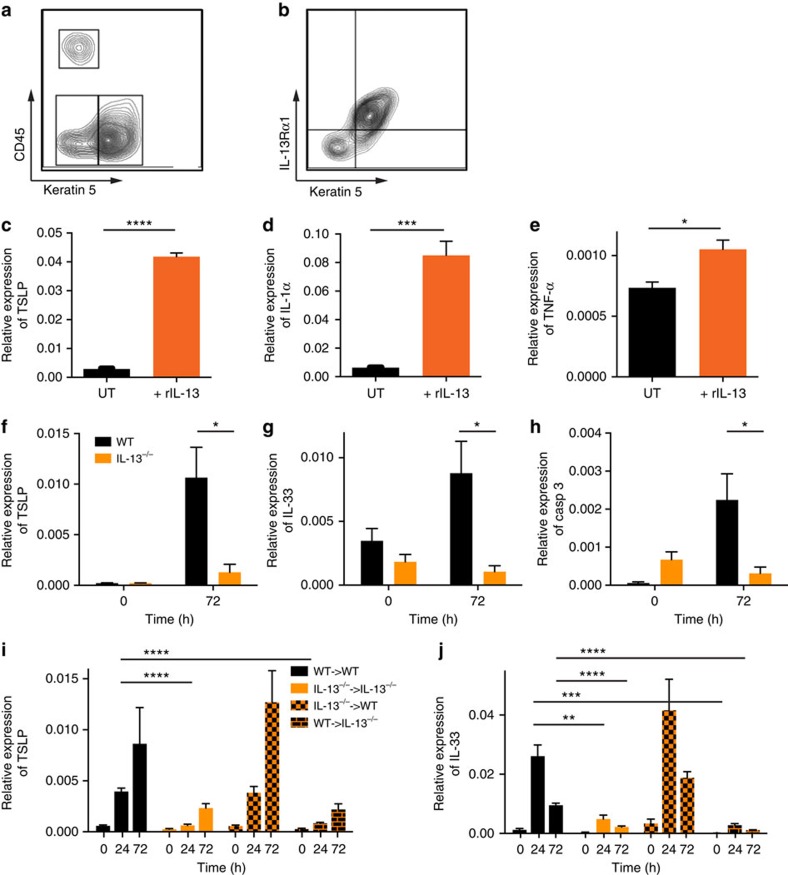
IEL-derived IL-13 activates skin EC. (**a**,**b**) Epidermal cell suspensions from resting WT skin stained for CD45, keratin 5 and IL-13Rα1. Representative examples shown. (**c**–**e**) Primary neonatal KCs cultured *in vitro* with/without 20 ng ml^−1^ rIL-13 were assessed for expression of (**c**) TSLP, (**d**) IL-1α and (**e**) TNFα (*n*=6). UT, untreated. (**a**–**e**) data from WT FVB mice. (**f**–**h**) WT and IL-13^−/−^ mice were treated topically *in vivo* with DMBA once and freshly isolated epidermis analysed by qRT–PCR 72 h later for expression of the genes (**f**) TSLP, (**g**) IL-33 and (**h**) caspase-3. (**i**,**j**) Fully reconstituted BM chimera mice were treated with a single skin exposure of DMBA (8 weeks after BM transplant) and isolated epidermis analysed for (**i**) TSLP and (**j**) IL-33 expression at the indicated time points (*n*=4 per time point). IL-13^−/−^→WT indicates that the donor is IL-13^−/−^ and the host WT (IL-13-sufficient IELs); WT→IL-13^−/−^ indicates that the donor is WT and the host IL-13^−/−^ (IL-13-deficient IELs). Data in **c**–**j** were done by qRT–PCR and are expressed as mean±1 s.e.m. relative to the control gene cyclophylin. WT mice are shown in black bars and IL-13^−/−^ mice in orange bars. (**f**–**j**) Data from mice on BALB/c background. Statistical significance of difference between experimental groups was determined using Student's *t*-test for unpaired data with *P<0.05, **P<0.01, ***P<0.001 and ****P<0.0001.

IL-13 mediates its effects by interacting with a complex receptor system comprised of IL-4Rα and two IL-13-binding proteins, IL-13Rα1 and IL-13Rα2 (ref. 18). At steady state in the resting skin, all the basal keratin 5^+^ KCs expressed the main IL-13Rα1 (Fig. 2a,b) and IL-4Rα1 and IL-13Rα2 were also expressed by KCs (Supplementary Fig. 4). An important mechanism of modulating IL-13 responses may be through the regulation of its receptors, and indeed following topical skin abrasion (Supplementary Fig. 4A,B) or exposure to DMBA (Supplementary Fig. 4C,D) both IL-4Rα1 and IL-13Rα1 were further upregulated. The regulation of the IL-13 receptors appeared to be independent of IL-13, as a similar upregulation on stressed KCs *in vivo* was seen in mice deficient in IL-13 (Supplementary Fig. 4C,D). The IL-13Rs on KCs were active as addition of recombinant (r)IL-13 to primary KCs *in vitro* induced further upregulation of IL-4Rα1 and particularly of the ‘decoy' receptor IL-13Rα2 (Supplementary Fig. 4E–G), as well as production of TSLP, IL-1α and TNFα (Fig. 2c–e). In parallel, IL-13 was essential for the canonical KC stress response *in vivo*, as IL-13-deficient mice exposed topically to the carcinogen DMBA did not show the induction of TSLP, IL-33 or caspase-3 seen in wild-type (WT) mice (Fig. 2f–h). To determine whether IELs were the source of this IL-13, we exploited the fact that skin IELs are radioresistant (Supplementary Fig. 5) and generated bone marrow (BM) chimeras, in which IELs were the only haematopoietic cells in the skin that could produce IL-13. This was achieved by reconstituting irradiated WT mice with BM from mice in which both IL-13 alleles had been replaced by egfp (IL-13^egfp/egfp^→WT); for brevity, IL-13^egfp/egfp^ mice are termed IL-13^−/−^ henceforth. As controls, we generated chimeric mice in which IELs were IL-13 deficient, but other haematopoietic cells were IL-13 sufficient, by reconstituting irradiated IL-13^−/−^ mice with WT BM (WT→IL-13^−/−^), as well as WT mice reconstituted with WT BM (WT→WT) and IL-13^−/−^ mice reconstituted with IL-13^−/−^ BM (IL-13^−/−^→IL-13^−/−^). The chimeric mice all had similar numbers of canonical IELs and these were egfp^+^ in IL-13^−/−^→IL-13^−/−^ and WT→IL-13^−/−^ chimeras in which radioresistant host IELs were attempting to produce IL-13, but not in chimeras containing WT IELs (Supplementary Fig. 5D,E). Production of IL-13 was upregulated further on stimulation of IELs with PMA and ionomycin (Supplementary Fig. 5E). Conversely, egfp^+^ cells were only found in other lymphoid tissues in chimeric mice reconstituted with IL-13^−/−^ BM. Topical exposure of the chimeric mice to DMBA showed that mice that lacked IL-13 in the hematopoietic system, but had IL-13-sufficient IELs, had a normal KC response to stress, as shown by the induction of TSLP and IL-33. In contrast, KCs from mice with IL-13-deficient IELs (but IL-13-sufficient hematopoietic cells) did not respond normally to DMBA-induced stress (Fig. 2i,j). We conclude that IEL-derived IL-13 is central for a canonical KC stress response, demonstrating the importance of an integrated local tissue response to acute environmental challenges.

### IEL and IL-13 sustain tissue health and restore integrity

**Figure 3:**
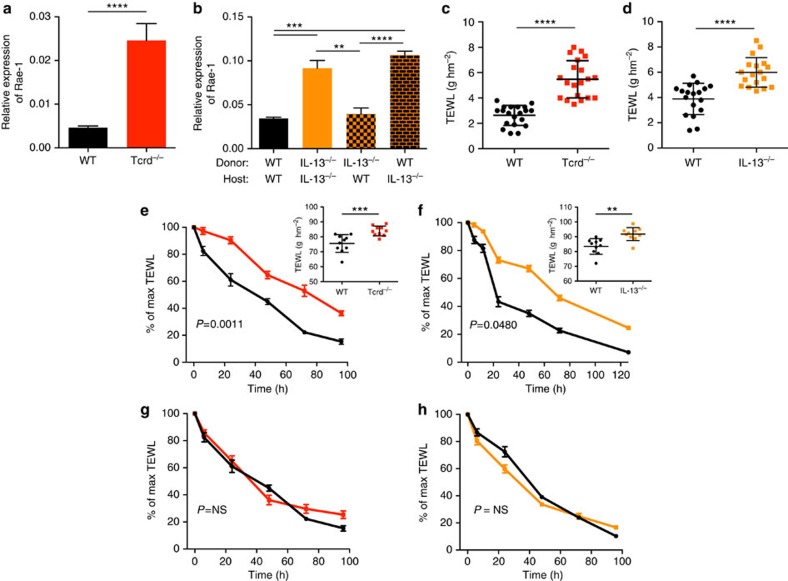
IELs and IL-13 maintain tissue health and restore integrity after insult. Rae-1 expression in the epidermis of naive (**a**) FVB WT (*n*=5), FVB Tcrd^−/−^ (*n*=5) and (B) WT BALB/c /IL-13^−/−^ BM chimeras (*n*=4 per group) was analysed by qRT–PCR. Data are expressed as mean±1 s.e.m. relative to the control gene cyclophilin. In **b**, IL-13^−/−^ donor to WT have IL-13-sufficient IELs; WT donor to IL-13^−/−^ host have IL-13-deficient IELs. (**c**–**h**) TEWL was analysed as a measure of barrier integrity using a tewameter probe assessing water evaporation rate and reported as g hm^−2^ (where g=water loss in grams, h=time in hours, m^2^=metres squared). TEWL was measured in naive (**c**) FVB WT and Tcrd^−/−^ mice (*n*=20 per group) as well as (**d**) BALB/c WT and IL-13^−/−^ mice (*n*=25–27 per group). (**e**,**f**) The dorsal ear skin was abraded by tape-stripping (6 ×) and TEWL measured just after tape-stripping and at indicated time points the following days in (**e**) FVB WT and Tcrd^−/−^ and (**f**) BALB/c WT and IL-13^−/−^ mice (*n*=10 in all groups). The data are expressed as % of the max TEWL measured just after tape-stripping. Max TEWL as measured just after tape-stripping is shown for all groups in the insert graphs. (**e**,**f**) were repeated twice with similar results. Black lines show WT groups, red line Tcrd^−/−^ and orange line IL-13^−/−^ mice. (**g**,**h**) The dorsal ear skin was abraded by tape-stripping (6 ×) and TEWL measured as before but (**g**) Tcrd^−/−^ and (**h**) IL-13^−/−^ mice were treated topically with 50 ng rIL-13 just after tape-stripping and again after 24 h (*n*=10 in all groups). (**g**) Red line shows Tcrd^−/−^ mice+rIL-13 and (**h**) orange line shows IL-13^−/−^ mice +rIL-13, WT mice treated with the vehicle, PBS, is shown in black lines. Statistical significance in **a**–**d** and inset graphs in **e**–**f** were determined using Student's *t*-test for unpaired data with **P<0.01, ***P<0.001 and ****P<0.0001. In **e**–**h**, statistical significance of difference between experimental groups was assessed over time using linear regression. NS, not significant.

To determine the relevance of IELs and the role of IL-13 on the health status of the epithelial tissue, we first looked at the resting steady-state skin. We found that the ECs appeared more stressed and had significant aberrant expression of the NKG2D ligand Rae-1 when canonical IELs were absent. This was evident also in total IL-13-deficient mice and even in mice where IL-13 was absent only in the IELs, while ECs in mice with IL-13-sufficent IELs had no or low expression of Rae-1 in spite of lacking IL-13 in the hematopoietic system (Fig. 3a,b). In addition, quantification of TEWL as a measure of skin integrity showed that even the resting skin had a small but significantly higher degree of water loss in the absence of canonical IELs or IL-13 (Fig. 3c,d). Moreover, when the skin of mice lacking canonical IELs or IL-13 was challenged by removing the stratum corneum by tape-stripping, an increased degree of water loss was observed, suggesting a worse barrier function and a poorer tolerance to damage (Fig. 3e,f, inserts). More strikingly, mice deficient in IELs or IL-13 showed a significantly impaired ability to repair the integrity of the barrier and re-establishing tissue homeostasis after the insult. The barrier was repaired much more slowly and even 5–6 days after insult, when WT skin was fully repaired, the mutants still showed a significant TEWL (Fig. 3e,f). Notably, this barrier-repair defect could be rectified by adding topically rIL-13 after the insult—both in mice lacking canonical IELs and in mice lacking IL-13 (Fig. 3g,h). Indeed, even topical application of IL-13 cytokine to WT mice after removal of the stratum corneum sped-up the recovery of an intact barrier (Supplementary Fig. 6A,B). However, for deeper skin insults beyond the epithelium, IL-13 was redundant in the repair of full skin thickness wounds (Supplementary Fig. 7), although closure of full-thickness skin wounds are significantly delayed in γδ T-cell-deficient mice^19^,^20^. Thus, skin IELs and their IL-13 production are vital in maintaining a healthy epithelium and play an important role in restoring epithelial tissue integrity following insult.

### IL-13 protects against cutaneous epithelial carcinogenesis

**Figure 4:**
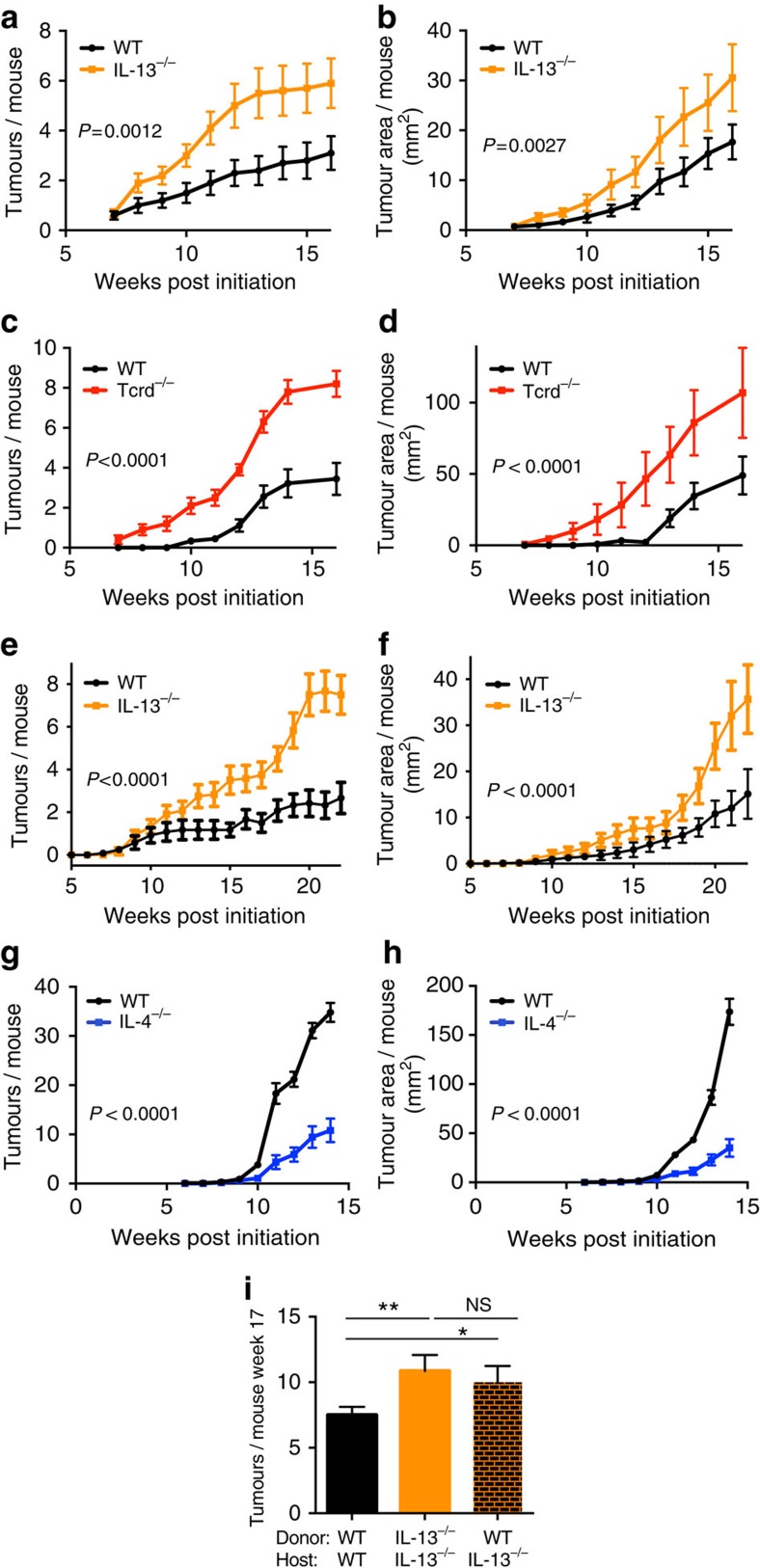
IELs and IL-13 protect against cutaneous epithelial carcinogenesis. (**a**–**d**) The shaved backs of mice were treated once weekly with 200 nmol DMBA to evoke ‘complete DMBA' carcinogenesis. Animals were scored, tumours counted and measured once weekly. Data are expressed as average number of tumours per (**a**) BALB/c WT and IL-13^−/−^ mouse, and (**c**) FVB WT and Tcrd^−/−^ mouse, and average tumour size per (**b**) BALB/c WT and IL-13^−/−^ mouse, and (**d**) FVB WT and Tcrd^−/−^ mouse (*n*=10–14 per group). (**e**–**h**) The shaved backs of mice were treated with a single subcarcinogenic dose of DMBA followed by twice weekly application of TPA to promote chronic skin inflammation and outgrowth of tumours. Mice were scored weekly and the data expressed as (**e**,**g**) average tumour number or (**f**,**h**) tumour size per (**e**,**f**) BALB/c WT or IL-13^−/−^ mouse (*n*=12 per group) or (**g**,**h**) FVB WT or IL-4^−/−^ mouse (*n*=10 per group). Statistical significance between groups was assessed over time using linear regression. (**i**) BALB/c WT/IL-13^−/−^ BM chimera mice were generated as described and 8 weeks later the mice were subjected to ‘DMBA complete' carcinogenesis. Data show the average number of tumours per mouse at the end of the experiment, week 17. WT donor to IL-13^−/−^ host have IL-13-deficient IELs (*n*=8 per group). Statistical significance of difference between experimental groups was determined using Student's *t*-test for unpaired data with *P<0.05 and **P<0.01. NS, not significant.

We have shown that the same IEL that is host-protective against inflammation-driven carcinogenesis is also a strong driver of type-2 immunity^9^,^12^ and local IL-13 responses (Fig. 1). Hence, we wanted to examine the role of IL-13 in epithelial carcinogenesis. We assessed skin tumour formation in IL-13-deficient mice using two different models of spontaneous epithelial carcinogenesis. First, we evoked ‘DMBA complete' carcinogenesis by exposing the back skin once weekly to the carcinogen DMBA. This does not cause inflammation in the tissue, but the ensuing accumulation of mutations in the ECs causes the outgrowth of tumours. Mice lacking IL-13 were significantly more susceptible to DMBA-induced carcinogenesis and developed both more and bigger tumours than WT mice (Fig. 4a,b). Mice deficient in canonical IELs were also highly susceptible to DMBA complete carcinogenesis, developing tumours earlier, as well as significantly more and bigger tumours than their WT counterparts (Fig. 4c,d). Next, we examined the commonly used model of DMBA–TPA carcinogenesis, where a single subcarcinogenic dose of DMBA, which causes EC mutations primarily in *hras* (initiation), is followed by repeated applications of the inflammatory reagent TPA (promotion), which allows for the few mutated clones to expand and form tumours. γδ T-cell-deficient and Vγ5Vδ1^+^ IEL-deficient mice are more susceptible to this form of two-stage chemical cutaneous carcinogenesis^9^,^15^. Consistent with this, mice lacking IL-13 were also more susceptible to this inflammation-driven carcinogenesis and developed significantly more and bigger tumours than WT controls (Fig. 4e,f). This is specific to IL-13 as mice lacking IL-4 were significantly less susceptible to tumour development following DMBA–TPA, suggesting that IL-4 may drive inflammation in this model, whereas IL-13 works differently (Fig. 4g,h). A constraint on this interpretation is the caveat that the IL-13 and IL-4 mutant mice used in this study were on a different background strain. There are well-known differences in susceptibility to DMBA–TPA carcinogenesis across different background strains^21^, making direct comparison between mutants challenging. All experiments on mutant strains were internally controlled with the appropriate background strain. Furthermore, using BM chimeric mice, we were additionally able to show that mice lacking IL-13 only in the IELs (WT→IL-13^−/−^) were as susceptible to DMBA carcinogenesis as mice completely lacking IL-13 and both mutants developed more tumours than WT (Fig. 4i). The increased susceptibility of IL-13^−/−^ mice to DMBA carcinogenesis was not due to an increased absorption of the carcinogen through the barrier-disrupted skin or an increased metabolism of DMBA due to differences in LCs or other skin DC populations (Supplementary Fig. 8), as the level of double-stranded DNA breaks in ECs caused by the initial DMBA exposure was similar between WT and IL-13^−/−^ mice (Supplementary Fig. 9). The tumour-protective effect of IL-13 additionally appears to be restricted to carcinogenesis at the epithelial skin barrier, as mice lacking IL-13 were not more susceptible to subcutaneous tumour growth (Supplementary Fig. 10). Together, this demonstrates that IL-13 is strongly protective against cutaneous epithelial carcinogenesis.

### IL-13 promotes EC maturation and transit through epidermis

**Figure 5:**
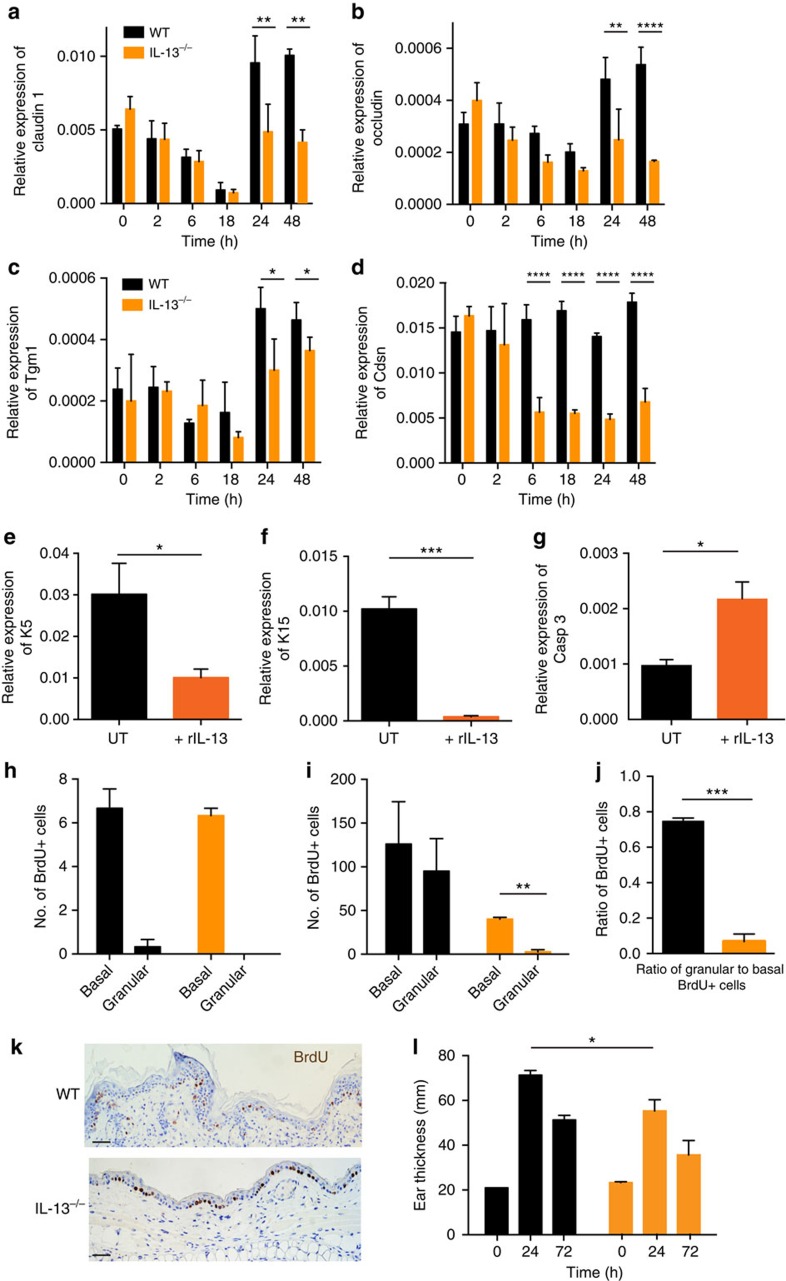
IL-13 promotes epithelial maturation and transit through the epidermis. Expression analysis of (**a**) claudin-1, (**b**) occludin, (**c**) transglutaminase 1 and (**d**) corneodesmosin in isolated epidermis from BALB/c WT mice (black bars) and IL-13^−/−^ mice (orange bars) at indicated time points after skin abrasion by tape-stripping (*nn*=3 per time point). (**e**–**g**) Primary neonatal KCs grown *in vitro* were stimulated with 20 ng ml^−1^ rIL-13 or left untreated (UT) and analysed for expression of the basal KC genes (**e**) Keratin 5 and (**f**) Keratin 15 as well as (**g**) caspase-3, which is associated with terminal KC differentiation (*n*=6). Data in (**a**–**g**) were done by qRT–PCR and are expressed as mean±1 s.e.m. relative to the control gene cyclophylin. (**h**–**k**) BALB/c WT (black bars) and IL-13^−/−^ (orange bars) were treated topically with 10 nmol TPA on the dorsal ear skin or left untreated (*n*=3 per group). After 21 h, they were injected with 200 μl BrdU solution and 3 h later ear skin was fixed in formalin, embedded in paraffin and BrdU staining performed on 5 μm sections. BrdU^+^ events were counted along the entire length of untreated and treated epidermis. Epidermal BrdU^+^ cells on the basal membrane were defined as ‘basal'; BrdU^+^ epidermal cells not on the basal membrane were defined as ‘granular'. Three 1-cm sections were counted per mouse. (**h**) Number of BrdU^+^ cells in the basal and granular epidermis at steady state without treatment and (I) 24 h after topical exposure to TPA. (**j**) The ratio of BrdU^+^ epidermal cells in the granular layer compared with the basal layer in BALB/c WT and IL-13^−/−^ mice. (**k**) Representative images of ear skin 24 h after exposure to TPA. Scale bar, 50 μm (**l**) Ear thickness was measured with calipers at 24 h and 72 h after a single skin exposure to 10 nmol TPA on the ear (*n*=6 per group). Data are represented as mean±1 s.e.m. Statistical significance of difference between experimental groups was determined using Student's *t*-test for unpaired data with *P<0.05, **P<0.01, ***P<0.001 and ****P<0.0001.

To explore how IL-13 promotes tissue integrity and protects against carcinogenesis, we studied its effect on epidermal structure and maintenance. The skin epithelium is under constant renewal. KCs move from the basal proliferative layer up through the spinal and granular layers undergoing differentiation and maturation before entering terminal differentiation and programmed cell death to form the cornified stratum corneum. To maintain tissue integrity and stability KCs express a multitude of structural proteins and complexes of scaffolding and adhesive proteins, which are tightly regulated during KC differentiation. In line with the barrier-repair defect that we found in the absence of IL-13 (Fig. 3), we also consistently found a reduced expression of tight junction proteins and structural components of the upper epidermis in IL-13-deficient mice when challenged *in vivo* to repair the barrier following tape-stripping (Fig. 5a–d). While BALB/c WT mice upregulated the expression of claudin-1, occludin and transglutaminase 1 at 24–48 h after the insult, in accordance with morphological tissue repair, this response was diminished in the absence of IL-13. FVB mice lacking epidermal IELs also showed diminished expression of claudin-1 and transglutaminase 1 after insult, but the kinetics of the response was slightly different on the FVB strain background (Supplementary Fig. 11). In addition, adding rIL-13 to primary KCs *in vitro* strongly downregulated genes associated with basal KCs such as K5 and K15 (Fig. 5e,f), suggesting that IL-13 promotes upward-moving KC maturation. This was supported by IL-13-induced expression of products associated with terminal differentiation such as caspase-3 both *in vivo* and *in vitro* (Figs 2h and 5g)^22^. To explore this further, we studied the rate of KC transit through the epidermis in a bromodeoxyuridine (BrdU) pulse-chase experiment. BrdU^+^ cells were counted in the basal epidermal layer along the basement membrane and compared with the number of BrdU^+^ cells in the granular layers; the role of IL-13 in this KC turnover and migration was evaluated at steady state and following topical exposure to TPA. In steady-state resting tissue, there were only few proliferating cells, these were nearly all in the basal layer—and there was no obvious difference between WT and IL-13-deficient mice (Fig. 5h). Following topical TPA application, many more KCs were proliferating and approximately half of these had moved into the granular layer in WT. However, in mice lacking IL-13, although more cells were proliferating than in the resting skin, these were nearly all in the basal layer and only very few had moved into the granular layers (Fig. 5i,k). This was particularly evident if the ratio of granular to basal BrdU^+^ cells was compared (Fig. 5j), illustrating that the KCs were migrating at a faster rate in IL-13-sufficient mice. As a result, the epidermis was also thinner in mice lacking IL-13 (Fig. 5k), consistent with previous reports showing that transgenic overexpression of IL-13 in basal KCs induces thickening of the epidermal layer^23^. Overall, the thickening of the total ear skin was reduced following topical TPA in IL-13-deficent mice (Fig. 5l). Together, this shows that the rate of EC movement from the basal layer is under control of IL-13 and hence IL-13 contributes to the upwards maturation of skin ECs and epithelial renewal.

This effect on EC transit time may possibly contribute to carcinogenesis susceptibility. In support of this, mice lacking canonical epidermal IELs had a higher level of mutant *hras* (A→T transversion within *hras* codon 61) in the epidermis following DMBA exposure but before clinical signs of disease, suggesting that the mutated cells accumulate in the tissue (Supplementary Fig. 12).

## Discussion

Immune surveillance can function by many (non-exclusive) mechanisms; it can recognize and remove damaged cells, remove or neutralize potential harmful environmental substances, facilitate re-establishment of homeostasis by tissue repair and dampen detrimental inflammation. Tissue-resident immunocytes are in a unique position to carry out a continued maintenance function such as immune stress surveillance and tissue-resident T cells can be direct afferent sensors of EC dysregulation^9^,^12^. This study provides insight into one mechanism, whereby γδ TCR^+^ IELs contribute to immune surveillance through direct action on neighbouring ECs, promoting tissue repair and protection against tumour formation. A general picture of an important role for γδ T cells, which are highly tissue-tropic, in cancer immune surveillance in both mouse and human is emerging. A recent large meta-analysis, integrating tumour gene expression with survival data, revealed the presence of intra-tumoral γδ T cells as the most significant favourable prognostic population of all leukocytes across human cancers^24^.

We show here that skin-resident γδ TCR^+^ IELs are potent producers of IL-13, following a variety of environmental stressors and this greatly affects EC function with pleiotropic consequences for tissue health. Human epidermal γδ T cells have likewise been shown to be high producers of type-2 cytokines—and particularly IL-13 (ref. 25). While much has been published on the effects of excessive expression of IL-4/IL-13 on the skin barrier in inflammatory skin diseases such as atopic dermatitis (AD), the potential role of constitutive expression of IL-13 has hitherto been less well understood. Both IELs, resident in the epidermis, and ILC2s, resident in the dermis, are constitutively positive for IL-13. ILC2s are increasingly acknowledged to be regulating cutaneous immune response^26^,^27^; however, they appear redundant in supporting acute EC responses in the epidermis. ILCs are ablated by radiation and re-populated by BM cells (data not shown and refs 26, 28), whereas IELs are radioresistant, so by generating BM chimeras we could reveal the important role of IEL-derived IL-13 for homeostatic responses in the epithelium. Overall, our study demonstrates that constitutive presence of IL-13 in the epidermis promotes barrier integrity and protects against carcinogenesis.

The regulation and function of type-2 immunity remain somewhat enigmatic. What is known so far of the physiological role of type-2 responses is that their host-protection properties converge in different forms of barrier defences^29^. Much of type-2 immunity appears dedicated to tissue repair and promoting tolerance to damage. Our data on IL-13 in the epidermis fits with this idea and is consistent with the previous data from γδ T-cell-deficient mice showing defects in the integrity of the epidermal barrier as well as a predisposition to develop spontaneous dermatitis^30^,^31^. A similar protective role for IL-13Rα1 signalling in ECs during lung injury and homeostasis has recently been reported^32^. This role for IL-13 in supporting ‘EC health' contrasts with the prevailing paradigm in AD, where the over-production of IL-13 is thought partly responsible for the abnormal epithelial barrier phenotype^33^,^34^. Type-2 cytokines are clearly upregulated in the barrier-disrupted skin in mice and in humans with AD^35^,^36^,^37^, but whether this is the cause or effect of skin disease has long been debated. In AD, most of the type-2 cytokines comes from the inflammatory infiltrate and it may well be that the ‘over-production' of IL-13 in this setting is too much/at the wrong time/from the wrong cells and is indeed detrimental for epidermal stability as studies from *in vitro* grown KC suggest^34^,^38^. However, as shown here, during acute insults, the homeostatic IL-13 response *in vivo* is clearly important for pushing basal KCs upwards and thereby aids the replenishing of the upper mature epidermis (Figs 5 and 3). This role for IL-13 as an ‘epithelial escalator' has previously been demonstrated in the gut epithelium, where it is thought that this is why IL-13 is essential for gut parasite expulsion^39^. Indeed, a recent study expands on this, and supports our described role of IL-13 in controlling EC fate decisions, by showing how IL-13 from intestinal ILCs regulate the cellular composition of the gut epithelium by signalling to uncommitted intestinal ECs^40^.

In terms of early cancer immune surveillance, the role of type-2 immunity has been little explored; the focus having been firmly on type-1 immunity and cytotoxic mechanisms, both of which have strong experimental support for playing a role in extrinsic tumour suppression. Nonetheless, the repair functions of type-2 immune surveillance are clearly important in protection against carcinogenesis, as illustrated by the link between wounding and tumour development^41^. Tumours can develop at the site of chronic skin wounds^42^,^43^ and patients with epidermolysis bullosa, who have chronic skin damage, are at increased risk of developing squamous cell carcinoma^44^,^45^. A diminished capacity to repair a damaged barrier can thus predispose to the development of cancer. Our data support this link, as deficiency in IL-13 resulted in both a barrier defect with reduced capacity to restore tissue integrity on insult and a significantly higher susceptibility to cutaneous carcinogenesis (Figs 3 and 4). A protective role for IL-13 in inflammation-driven skin carcinogenesis has previously been reported^46^ and ‘atopic' mice have likewise been shown to be protected^36^. Cipolat *et al.* showed that mice with a genetic barrier defect had an exaggerated type-2 response, including IL-13 and TSLP, and were protected against DMBA–TPA carcinogenesis. The ‘atopic' phenotype could be inhibited by blocking TSLP or NKG2D^36^, suggesting that LSS responses are induced by barrier disruption and protect against carcinogenesis also in this model. A homeostatic IL-13 response thus ensures normal tissue integrity (Fig. 3), but enhanced responses can induce hyperplasia and thickening of the epidermis (Fig. 5), which may primarily be driven by TSLP^47^. Acute hyperplasia results in improved resistance to damage and damaging substances at the body barrier and may therefore be another protective mechanism against carcinogenesis. The data presented here, showing a protective role for IL-13 in different models of epithelial carcinogenesis, provides new perspective to further studies on the links between atopy and cancer.

The association between defective tissue repair and cancer development may not only pertain to the lack of repair *per se* but also to the detrimental inflammation caused by chronic wounds. There is a close link between chronic inflammation and cancer, and once a malignant cell has escaped the early phase of immune surveillance, inflammation can exert prominent pro-carcinogenic effects^48^. An important component of early tumour surveillance could hence be the release of anti-inflammatory products in the tissue. IL-13 can have direct anti-inflammatory effects in the epithelial tissue as shown in the intestinal epithelium^49^, but recently a lot of focus has been on the more newly discovered EC cytokines—IL-33, TSLP and IL-25. Skin ECs promptly produce particularly IL-33 and TSLP upon stress, as when exposed to the carcinogen DMBA. Interestingly, this canonical EC stress response is under control of IEL-derived IL-13 (Fig. 2f–j). TSLP has been implicated in the development and progression of allergic diseases, in both human and mouse, in many recent studies and is thought to be a central regulator of atopy in several epithelial tissues^50^. TSLP is, however, also emerging as a potent tumour suppressor^51^. In the skin, TSLP is strongly protective against carcinogenesis^52^,^53^ and this is thought to be due to EC-derived TSLP perturbing the balance of inflammation in the tissue^53^. Clearly, not all type-2 immunity is anti-inflammatory. In this study, we show an interesting divergent effect of the type-2 cytokines IL-4 and IL-13 in inflammation-driven carcinogenesis. While IL-13 is protective, IL-4 clearly promotes tumour growth in this model (Fig. 4) and is mainly produced by the inflammatory infiltrate. It is interesting that, although these cytokines share many regulatory elements and have partially shared receptors, their expression pattern is often different^54^ and they have previously been shown to play distinct roles in asthma^55^. Indeed, the spatial and temporal expression of these divergent cytokines may also be very important in AD and further mechanistic studies are warranted to understand the role of type-2 immunity in regulating skin barrier function.

The fact that IELs and IL-13 protect against epithelial carcinogenesis, in both mutation- and inflammation-driven models, suggest that, in contrast to IL-4, this IEL–EC axis of communication plays a central regulatory role in addition to suppressing inflammation. This may be due to the role of IELs and IL-13 in controlling the rate of EC movement through the epidermis. IL-13 accelerates EC turnover and transit towards the upper epidermis. An increased ‘transit time' of skin ECs from the basal proliferative layer to the outer cell layers have previously been shown to be tumour suppressive in skin carcinogenesis^56^. Similarly, mice lacking the major structural protein keratin 10 in the upper epidermis have a strongly accelerated EC turnover and are significantly protected against skin carcinogenesis, suggesting an increased elimination of initiated KCs^57^. Conversely, skin cells resistant to terminal differentiation have since long been associated with initiation of carcinogenesis^58^.

In summary, we demonstrate that IL-13 acts as a molecular bridge between the skin IELs and ECs. This cross-communication regulates EC function and promotes skin homeostasis in a manner that protects against acute challenge and carcinogenesis. LSS is thus part of an early ‘allergic' type-2 host-defence mechanism aimed at protecting body surface tissues. A similar role for IL-13 in EC protection and homeostasis in the gut^39^,^40^ and lung^32^,^59^,^60^ raises the possibility that this may represent a conserved response against noxious environmental substances and damage to ECs—with potential important implications for atopy and cancer.

## Methods

### Mice

IL-13-egfp reporter mice were generated as described^61^ and bred to homozygosity for use as IL-13^−/−^. IL-13-egfp mice were on BALB/c strain background. *Tcrd*^−*/*−^ mice^62^ and IL-4^−/−^ were backcrossed onto FVB/N background >10 generations. BALB/c and FVB/N WT mice were purchased from Charles River and used as controls. For experiments requiring neonatal KCs, neonatal mice were bred in-house. Mice were bred and maintained in individually ventilated cages under specific pathogen-free conditions; with food and water provided *ad libitum*. Age-matched, female mice were used for all experiments at ≥7 weeks of age. All studies were approved by Imperial College AWERB (Animal Welfare and Ethical Review Body) and the UK Home Office for Laboratory Animal Care regulations. Experiments involving cancer studies strictly adhered to the guidelines set out by the National Cancer Research Institute (NCRI) and Workman *et al.*^63^ in ‘Guidelines for the Welfare and Use of Animals in Cancer Research', and all studies using animals were conducted following the Animal Research: Reporting In Vivo Experiments (ARRIVE) guidelines^64^.

### BM chimeras

Mice were sub-lethally irradiated (750 rad) and immediately reconstituted with 5 × 10^6^ donor BM cells intravenous. Mice were left for 8 weeks, to fully reconstitute the hematopoietic system, and thereafter the reconstitution was examined by flow cytometry (Supplementary Fig. 2). Chimeric mice were used for experiments at ≥8 weeks after BM reconstitution.

### Tissue processing

Ears were collected and split into dorsal and ventral sides. For isolation of intact epidermal sheets, the skin was floated dermal side-down in 0.5 M NH_4_SCN for 40 min, 37 °C, 5% v/v CO_2_. Epidermal sheets were gently lifted away from the dermis and washed in PBS. The fixed epidermal sheets were then processed either for RNA extraction or for microscopy. Reagents and staining method for epidermal sheets can be found in the Supplementary Information.

For isolation of epidermal single cell suspensions, the skin was floated dermal side-down in filter-sterilized, TrypLE Express (Life) solution for 2 h at 37 °C, 5% v/v CO_2_. Following digestion, epidermis was separated from the dermis and further digested in TrypLE Express solution supplemented with 200 μg ml^−1^ DNAse I (Roche) and 1 × DNAse buffer (1.21 g l^−1^ Tris Base, 0.5 g l^−1^ MgCl_2_ and 0.073 g l^−1^ CaCl_2_) on a rotator for 30 min at 37 °C. Cell suspensions were filtered and washed in PBS before flow cytometry staining.

### Flow cytometry staining

Cell suspensions were stained with a fixable, live/dead discrimination dye (Life) and subsequently blocked for non-specific binding using antibody against FcγR (2.4G2) and 2% normal rat serum (Sigma). For staining of cell surface markers, cell suspensions were stained with fluorochrome-conjugated antibodies and appropriate isotype controls for 40 min and subsequently washed. For intracellular cytokine staining cells were incubated for 4 h with brefeldin A at 10 μg ml^−1^, with or without PMA and ionomycin (6.25 ng ml^−1^ and 3.5 μg ml^−1^, respectively). Following stimulation, cells were washed in PBS, resuspended and fixed/permed with Fixation and Permeabilization Buffer Set (Affymetrix, CA, USA) before antibody labelling. For intranuclear γH2AX staining, cells were fixed/permed in ice-cold 70% ethanol at −20 °C for 2 h, then blocked with 2% normal mouse serum, Fc-block and 2% fetal calf serum for 15 min, followed by 45 min staining for γH2AX at room temperature. Stained cells were analysed immediately using a BD FACSVerse (BD Biosciences, NJ, USA) machine. Data analysis was performed using FlowJo 10 for Mac (TreeStar, OR, USA). Antibodies were sourced from eBioscience unless otherwise stated. The following antibodies were used: CD45 (30-F11), Cytokeratin 5 (D516B4; Millipore), IFN-γ (XMG1.2), IL-13 (eBio13A), IL-13 Rα1 / CD213α1 (13MOKA), TCR Vγ5 (536; BioLegend), γH2AX (JBW301; Millipore), CD64 (X54-5/7.1; BioLegend), CD11c (HL3), CD11b (M1/70), CD207 (4C7; BioLegend), CD103 (2E7) and XCR1 (ZET; BioLegend).

### Epidermal sheet immunofluorescence

Epidermal sheets were fixed in ice-cold acetone at −20 °C for 15 min, then rehydrated in PBS and blocked with 2% bovine serum albumin. Sheets were then stained with a fluorescein isothiocyanate-conjugated anti-Vγ5 or anti-Langerin antibody and subsequently washed thoroughly in PBS. Sheets were carefully mounted onto slides using anti-fade mounting medium (Dako) and visualised on a Leica SP5 (Leica) confocal microscope.

### Immunohistochemistry

A 1 × 1-cm skin cut from the centre of the ears were fixed in formalin, embedded in paraffin and 5-μm sections cut. Slides were de-waxed, antigen retrieval was performed with pH 6.0 sodium citrate treatment at 95 °C for 15 min, endogenous tissue peroxidase activity was blocked with H_2_O_2_ treatment, and non-specific binding was blocked with normal serum and fish skin gelatin (Sigma). Non-specific avidin and biotin-binding sites were blocked with an Avidin/Biotin blocking kit (VECTASTAIN). For BrdU detection, slides were stained with anti-BrdU antibody (clone BU1/75; Abcam) in conjunction with a three-layer immunohistochemistry kit (VECTASTAIN *Elite* ABC kit; Vectastain) as per the manufacturer's instructions. Staining was visualised with DAB substrate and brown chromogen precipitation. Slides were counter stained in Harris' haematoxylin acid solution, dehydrated and mounted with Pertex mouting. Visual inspection of tissue and counting of BrdU^+^ cells were performed while blinded to the experimental groups.

### qRT–PCR and primer sequences

RNA was extracted from ammonium-fixed epidermis with an RNEasy Mini kit (Qiagen). RNA was dissolved in nuclease-free water, and yield and purity were determined. Complementary DNA (cDNA) was synthesised from RNA with a iScript cDNA synthesis kit (Bio-Rad) as per the manufacturer's instructions. cDNA was diluted in nuclease-free double-deionized water for qRT–PCR. All primers were single-stranded DNA oligonucleotides (Sigma) that were intron-spanning as verified by NCBI Primer-Blast tool. Real-time PCR product was detected with SYBR Green (Life) measured continuously with a ViiA 7 Real-Time PCR system (Applied Biosystems, CA, USA). Ct values for genes of interest were normalised against Ct values of the housekeeping gene Cyclophilin (*Cyc*) using the 2^−ΔCt^ method.

The following primers were used. F denotes forward primer (5′–3′) and R denotes reverse primer (3′–5′). *Il13* (F: 5′-GCTTATTGAGGAGCTGAGCAACA-3′, R: 5′-GCCAGGTCCACACTCCATA-3′); *Il4ra1* (F: 5′-CCAATCAGACAGATACCAGATG-3′, R: 5′-CCAGGTCAGCAGCCATTC-3′); *Il13ra1* (F: 5′-AGAGGTTGAAGAGGACAAATGCC-3′, R: 5′-GCGACAAAGACTGGAATGGTGAG-3′); *Il13ra2* (F: 5′-CCGAAATGTTGATAGCGACAGC-3′, R: 5′-CCAAGCCCTCATACCAGAAAAAC-3′); *Tslp* (F: 5′-TCGAGGACTGTGAGAGCAAG-3′, R: 5′-TGTTTTGTCGGGGAGTGAA-3′); *Il1a* (F: 5′-TTGGTTAAATGACCTGCAACA-3′, R: 5′-GAGCGCTCACGAACAGTTG-3′); *Tnfa* (F: 5′-ACTGGAGTTGTACGGCAGTG-3′, R: 5′-GGCTGATCCCGTTGATTTCC-3′); *Casp3* (F: 5′-GAGCTTGGAACGGTACGCTA-3′, R: 5′-5′-GCGAGATGACATTCCAGTGC-3′); *Il33* (F: 5′-CACATTGAGCATCCAAGGAA-3′, R: 5′-AACAGATTGGTCATTGTATGTACTCAG-3′); *Rae1* (F: 5′-TGGACACTCACAAGACCAATG-3′, R: 5′-CCCAGGTGGCACTAGGAGT-3′); *Claudin1* (F: 5′-5′-GCCATCTACGAGGGACTGTG-3′, R: 5′-5′-CACTAATGTCGCCAGACCTGAA-3′); *Occludin* (F: 5′-TTGAACTGTGGATTGGCAGC-3′, R: 5′-CAAGATAAGCGAACCTTGGCG-3′); *Tgm1* (F: 5′-CCTTGAGCTCCTCATTGGAA-3′, R: 5′-CCCTTACCCACTGGGATGAT-3′); *Cdsn* (F: 5′-AATGTCCAGCCCGGCATAAA-3′, R: 5′-CAAGATTCCTGGCAGAATAAGACC-3′); *K5* (F: 5′-CATGTCTCGCCAGTCCAGTG-3′, R: 5′-GGAACCGCACCTTGTCGATG-3′); *K15* (F: 5′-5′-GGAAGAGATCCGGGACAAA-3′, R: 5′-TGTCAATCTCCAGGACAACG-3′); and *Cyc* (F: 5′-CAAATGCTGGACCAAACACAA-3′, R: 5′-CCATCCAGCCATTCAGTCTTG-3′).

DMBA-induced transversion mutation in *hras* (codon 61, CAA→CTA) was quantified with a forward primer, mutant-specific reverse primer and a custom generated TaqMan 6′-FAM-conjugated probe for visualizing product: hras C61^CAA→CTA^ (F: 5′-CTAAGCCTGTTGTTTTGCAGGAC-3′, R: 5′-CATGGCACTATACTCTTCTA-3′; Probe: 5′-6FAM-CGGAAACAGGTGGTCAT-MGB-3′).

### Primary neonatal KC cultures

Body wall skin from neonatal mice was incubated overnight at 4 °C in 5 U ml^−1^ Dispase (BD) solution supplemented with 1 × antibiotic and antimycotic solution (Sigma). The epidermis was isolated and further digested in TrypLE Express solution supplemented with 200 μg ml^−1^ DNAse I and DNAse buffer. Cell suspensions were filtered, resuspended in defined KC serum-free medium with supplements (Life)+1 × antibiotic–antimycotic solution. KCs were seeded at an appropriate cell density onto tissue culture vessels coated with rat-tail-derived-collagen I (Sigma). Culture vessels were washed with PBS 24 h following seeding to remove unattached cells, and provided with fresh medium±20 ng ml^−1^ recombinant IL-13 (R&D). For extraction of KCs, cell culture vessels were treated with trypsin and KC RNA was subsequently extracted using the RNEasy Mini kit (Qiagen).

### Tape-stripping and TEWL

The stratum corneum was removed from the ear skin by application and removal of cellophane tape (Scotch) six times per ear. For measurement of TEWL, a tewameter probe (Tewameter TM300; Ck Electronic, Germany) was placed directly onto the ear skin of anaesthetized mice in a temperature- and humidity-controlled facility. Probe readings were analysed and water evaporation rate was reported using the MPA software (MPA software; Ck Electronic, Germany) as g hm^−2^ (where g=water loss in grams, h=time in hours, m^2^=metres squared). For some experiments, mice were treated topically on the ear skin with 50 ng rIL-13 (R&D), or vehicle control, immediately following tape-stripping, and again 24 h later.

### Chemical cutaneous carcinogenesis

The dorsal back area was shaved with a surgical blade and mice rested for 1 week. Applications of chemicals and tumour monitoring were performed as previously described^15^,^65^. Chemicals, in a 100 μl volume, were carefully and slowly applied by pipette to the entire shaved skin area. For ‘DMBA complete' carcinogenesis, mice were treated once weekly with 200 nmol DMBA. For two-stage ‘DMBA+TPA' carcinogenesis, mice were initiated with 200 nmol DMBA followed by twice weekly application of 10 nmol (FVB background) or 20 nmol (BALB/c background) TPA. A slightly different dosage between the background strains was chosen due to the well-known difference in susceptibility to chemical skin carcinogenesis across strains^21^. Despite this, the FVB background remained far more susceptible and developed a higher tumour load. All experiments on mutant mice were controlled with the appropriate background strain. Hair regrowth during experiments was gently removed by clipping with trimmers. Mice were monitored daily and cutaneous tumours were counted and measured with a caliper once weekly. Back skin and tumours were evaluated by visual inspection by an observer blinded to the experimental groups.

### Full skin thickness wounding

Mice were anaesthetized with inhalation isoflurane and transferred onto a heat block with an anaesthetic nose-cone to maintain anaesthesia throughout the procedure. Buprenorphine local analgesic (Vetergesic) was administered subcutaneously around the area to be wounded. A single, full-thickness 5-mm wound was made at the midline of the lower back using a disposable sterile punch biopsy tool. Sterile paper bedding and wet food were provided for 7 days following wounding. Wound size was measured daily with calipers for the first 2 days, and then once every 2 days until complete wound closure. The wound sagittal (*x*) and transverse (*y*) plane were measured and these were applied to the ellipse area formula to calculate wound area: (area=*π* (radius *x*)(radius *y*)).

### Subcutaneous tumour inoculation

The right flank of mice was shaved and 1 × 10^4^ CT26 cells injected subcutaneously into the flank in 100 μl sterile PBS. The mice were inspected daily, the flank was palpated and the tumour mass was measured with calipers.

### Statistical evaluation

The statistical significance of difference between experimental groups was determined using two-tailed Student's *t*-test for unpaired data or linear regression, where appropriate, with results deemed significant at *P*<0.05. Stars of significance correlate to: *P<0.05; **P<0.01; ***P<0.001; ****P<0.0001. No mice were excluded from analysis. Statistics was performed with GraphPad Prism 6.00 for Mac (GraphPad; La Jolla, CA, USA).

### Data availability

The data that support the findings of this study are available from the corresponding author upon request.

## Additional information

**How to cite this article:** Dalessandri, T. *et al.* IL-13 from intraepithelial lymphocytes regulates tissue homeostasis and protects against carcinogenesis in the skin. *Nat. Commun.* 7:12080 doi: 10.1038/ncomms12080 (2016).

## Supplementary Material

Supplementary Information Supplementary Figures 1-12.
