# Supplementary material for: IL-13 from intraepithelial lymphocytes regulates tissue homeostasis and protects against carcinogenesis in the skin
Source: Nat Commun. 2016 Jun 30;7:12080. doi: 10.1038/ncomms12080 (PMC4931319; doi:10.1038/ncomms12080)
Supplement: Supplementary Information — Supplementary Figures 1-12. [file ncomms12080-s1.pdf]

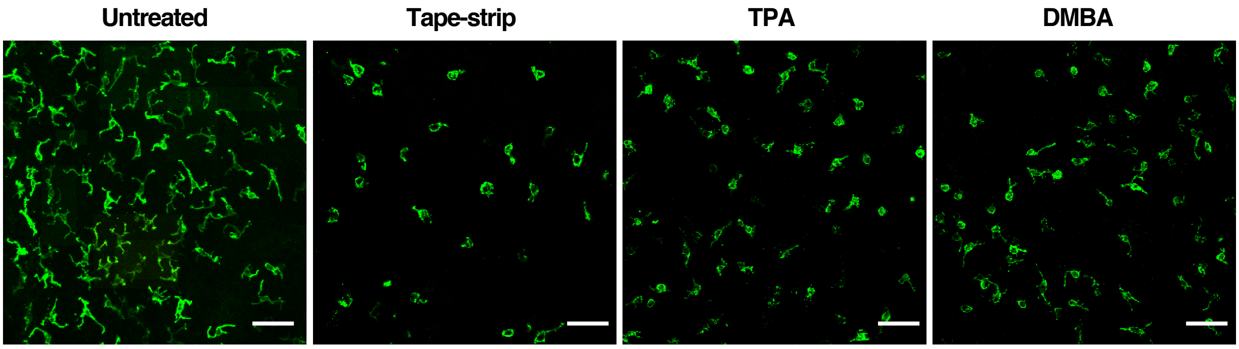

Supplementary Figure 1. Epidermal IELs are responsive to topical environmental challenge

Confocal microscopy of IELs examining their morphology *in situ* in epidermal sheets freshly isolated from the ear of FVB WT mice (from left to right) untreated, abraded by tape-stripping, exposed topically to TPA or DMBA. Epidermis was isolated 24h after skin challenge and skin IELs visualized with Vγ5 TCR ab (green). Representative images are shown. Original magnification x40. Scale bars = 50μm.

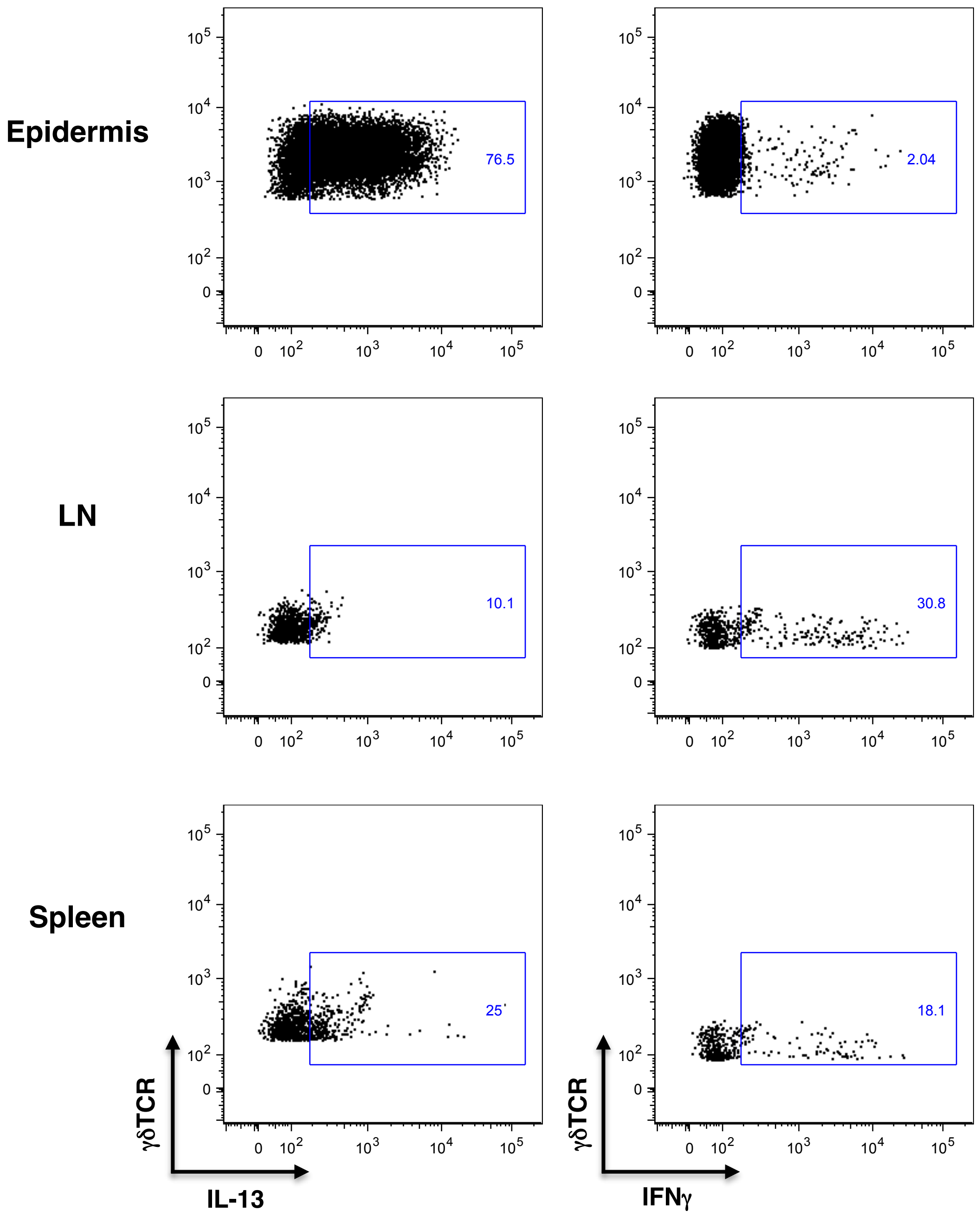

Supplementary Figure 2. Epidermal γδ T cells are uniquely biased towards IL-13 production

IL-13 and IFNγ production by γδ T cells isolated from the epidermis, skin draining lymph node (LN) and spleen from the same FVB WT animal was analysed following 4h *in vitro* stimulation with PMA/ionomycin. Cells were stained and gated on the γδTCR and intracellular levels of IL-13 and IFNγ assessed by FACS. Cytokine positive cells were defined using isotype control gating. Representative examples are shown.

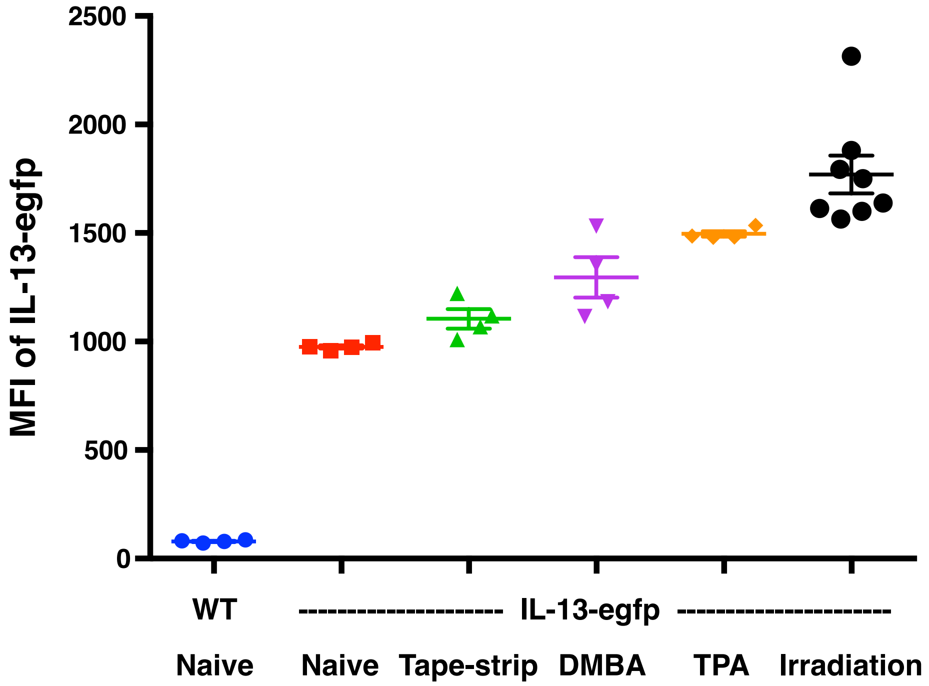

Supplementary Figure 3. Epidermal IELs express IL-13 in resting skin and upregulate expression further following challenge

Skin γδ T cells were isolated from resting naïve WT BALB/c and IL-13-egfp reporter mice as well as from IL-13-egfp mice following challenge by topical abrasion (tape-stripping), topical exposure to the carcinogen DMBA or to TPA and following whole body irradiation (750 Rads). IL-13-egfp MFI was subsequently assessed by FACS (n=4-8 per group). All epidermal γδ TCR^+ IELs were positive for IL-13-egfp already at resting state (100%) with a mean MFI of 975. The mean IEL egfp MFI was further enhanced following challenge ($p<0.05$ for tape-stripping and DMBA, $p<0.0001$ for TPA and irradiation). All dermal γδ T cells remained egfp negative throughout.

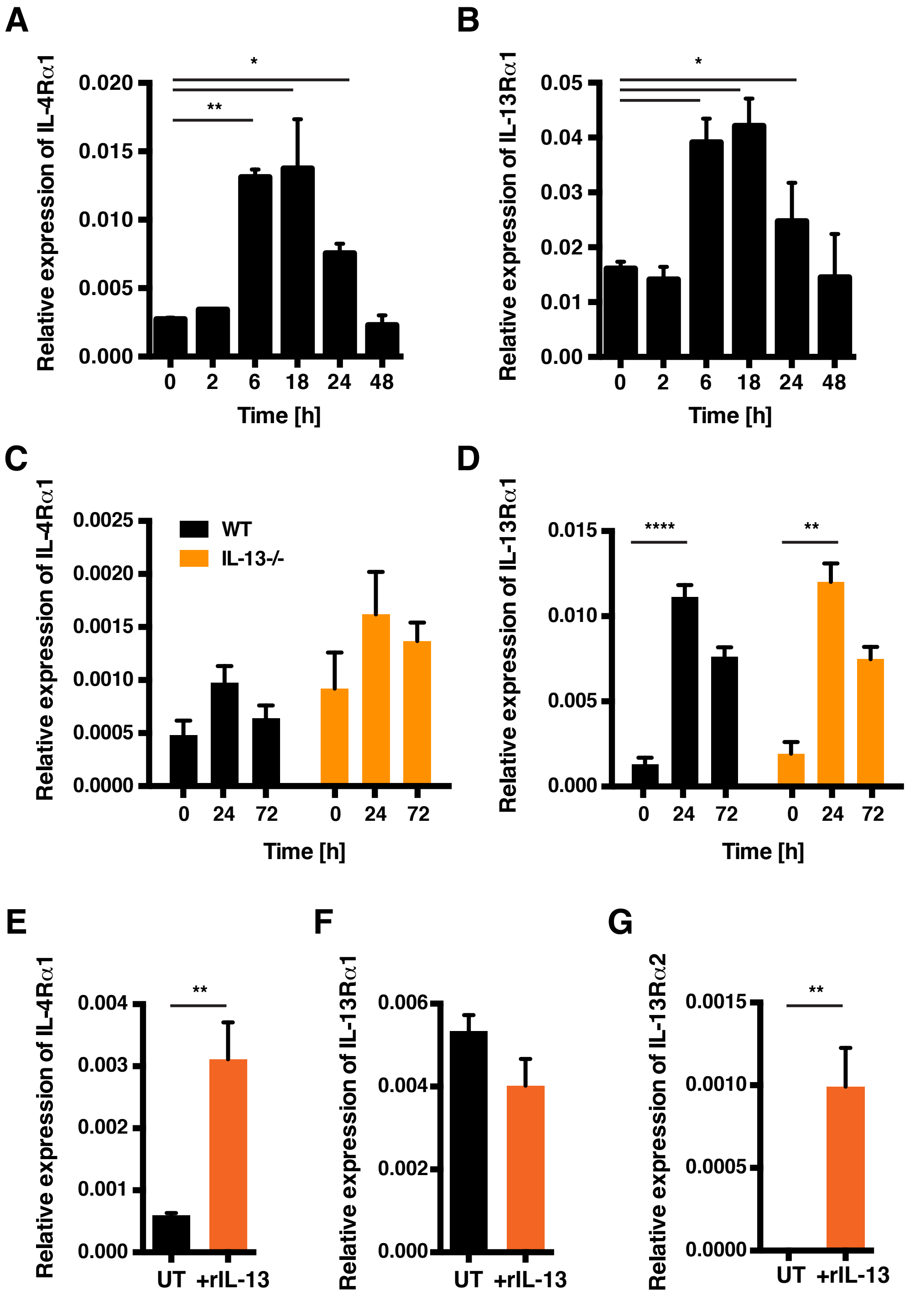

Supplementary Figure 4. IL-13R expression is stress-regulated on KCs
Quantitative RT-PCR analysis of (A) IL-4αR1 and (B) IL-13αR1 mRNA in isolated FVB WT epidermis at the indicated time-points after skin abrasion by tape-stripping (n=3 per time-point). (C-D) Expression of IL-4αR1 and IL-13αR1 mRNA in epidermis of BALB/c WT and IL-13$^{-/-}$ mice at indicated time-points after a single skin exposure to DMBA (n=4 per time-point). (E-G) Primary neonatal KCs grown *in vitro* were assessed for expression of (E) IL-4αR1, (F) IL-13αR1 and (G) IL-13αR2 receptor with/without stimulation by 20 ng/ml rIL-13 (n=6). UT = untreated. Data in (A-C) was done by qRT-PCR and are expressed as mean ± 1 SEM relative to the control gene cyclophylin. BALB/c WT mice are shown in black bars, IL-13$^{-/-}$ mice in orange bars. Statistical significance of difference between experimental groups was determined using Student's t-test for unpaired data with * correlating to $p<0.05$, ** $p<0.01$, *** $p<0.001$ and **** $p<0.0001$.

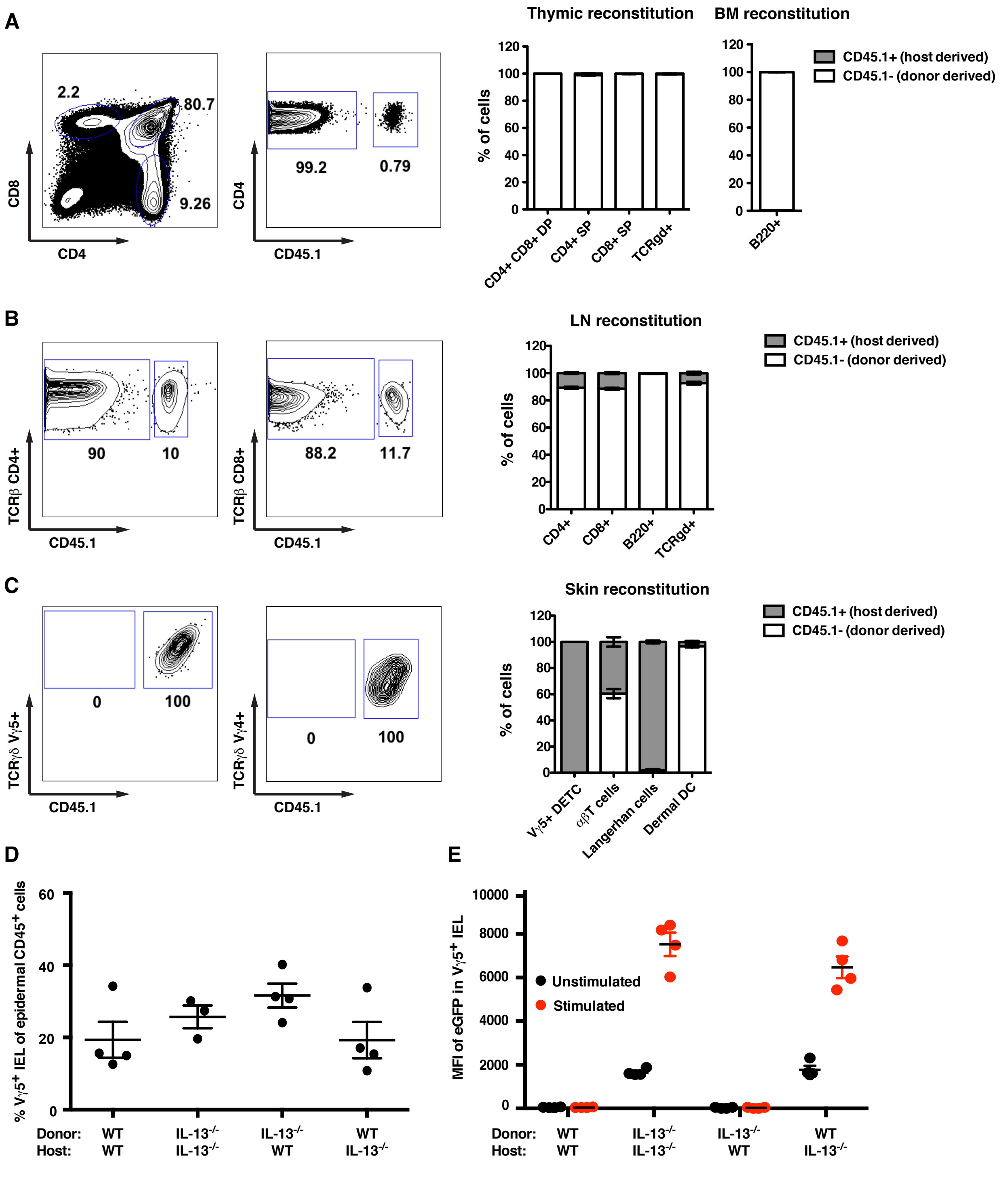

Supplementary Figure 5. IL-13-sufficient or IL-13-deficient IELs are created by BM chimerism
(A-C) $CD45.1^+$ host mice were full-body radiated at 750rads and immediately transplanted with $CD45.1^-$ donor bone marrow (BM). 8 weeks after the BM transplant organs and tissues were analysed for host ($CD45.1^+$) cells versus donor ($CD45.1^-$) cells in (A) thymus and BM B cells, (B) peripheral LN and (C) skin. (D-E) BALB/c WT/IL-$13^{-/-}$ BM chimeras were created by reconstituting irradiated WT mice with BM from mice in which both IL-13 alleles had been replaced by egfp (IL-$13^{egfp/egfp}$→WT). (For brevity IL-$13^{egfp/egfp}$ mice are termed IL-$13^{-/-}$ henceforth). As controls, irradiated IL-$13^{-/-}$ mice were reconstituted with WT BM (WT→IL-$13^{-/-}$), WT mice reconstituted with WT BM (WT→WT) and IL-$13^{-/-}$ mice reconstituted with IL-$13^{-/-}$ BM (IL-$13^{-/-}$→IL-$13^{-/-}$). (D) Number of $V\gamma5^+$ skin IELs were assessed in the epidermis 8 weeks after reconstitution and (E) their egfp signal assessed by flow cytometry at resting or following *ex vivo* stimulation with PMA/iono (n=4 per group). IL-$13^{-/-}$ donor to WT have IL-13-sufficient IELs; WT donor to IL-$13^{-/-}$ host have IL-13-deficient IELs.

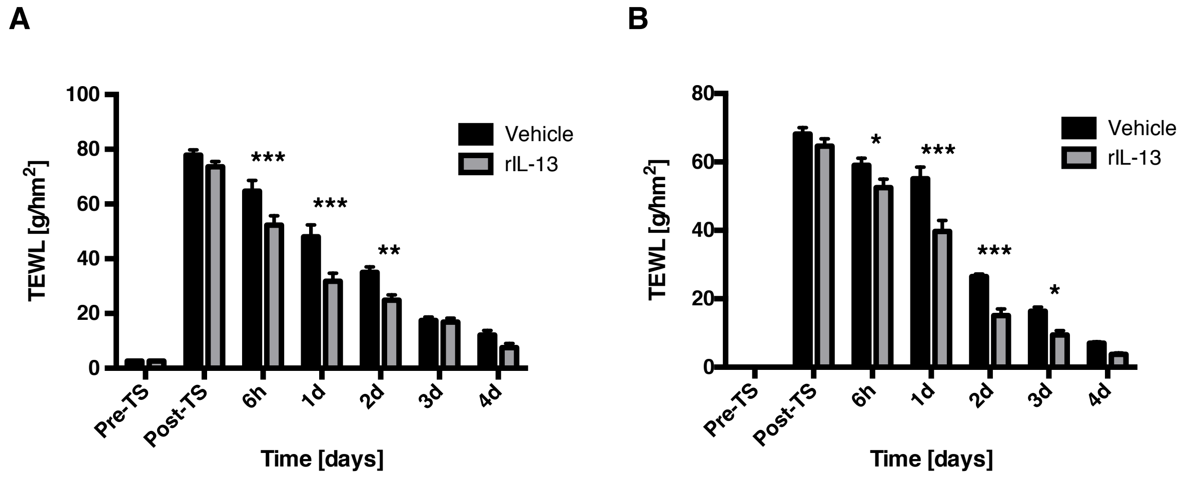

Supplementary Figure 6. rIL-13 administered topically enhances speed of barrier recovery

TEWL was measured on the dorsal ear before and at various times after skin abrasion by tape-stripping (TS) (6x). (A) FVB WT mice were treated topically with 50ng rIL-13 just after tape-stripping and again after 24h and compared to mice treated with vehicle only (PBS) (n=10 per group). (B) BALB/c WT mice were treated topically with 50ng rIL-13 just after tape-stripping and again after 24h and compared to mice treated with vehicle only (n=8 per group). Statistical significance of difference between experimental groups was determined using Student's t-test for unpaired data with * correlating to $p<0.05$, ** $p<0.01$ and *** $p<0.001$.

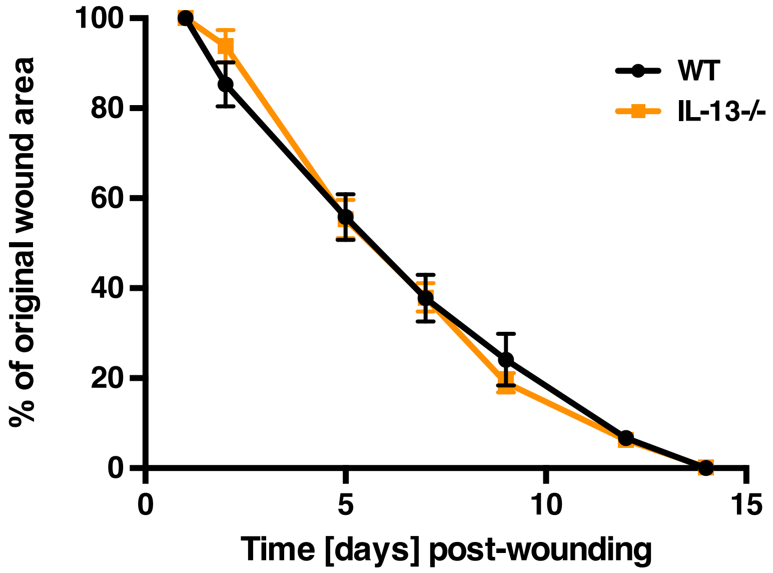

Supplementary Figure 7. IL-13 is redundant for full skin thickness wound healing
A full skin thickness, 0.5 mm punch biopsy, wound was made on the lower back of BALB/c WT and IL-$13^{-/-}$ mice and wound closure assessed over time. Wound sizes were measured with calipers daily for the first 2 days, and then once every 2 days until complete wound closure. The wound sagittal (*x*) and transverse (*y*) plane was measured and these were applied to the ellipse area formula to calculate wound area: (area = π (radius *x*)(radius *y*)). Data is shown as mean % of the original wound area at day 0 ± 1 SEM (n=8 per group).

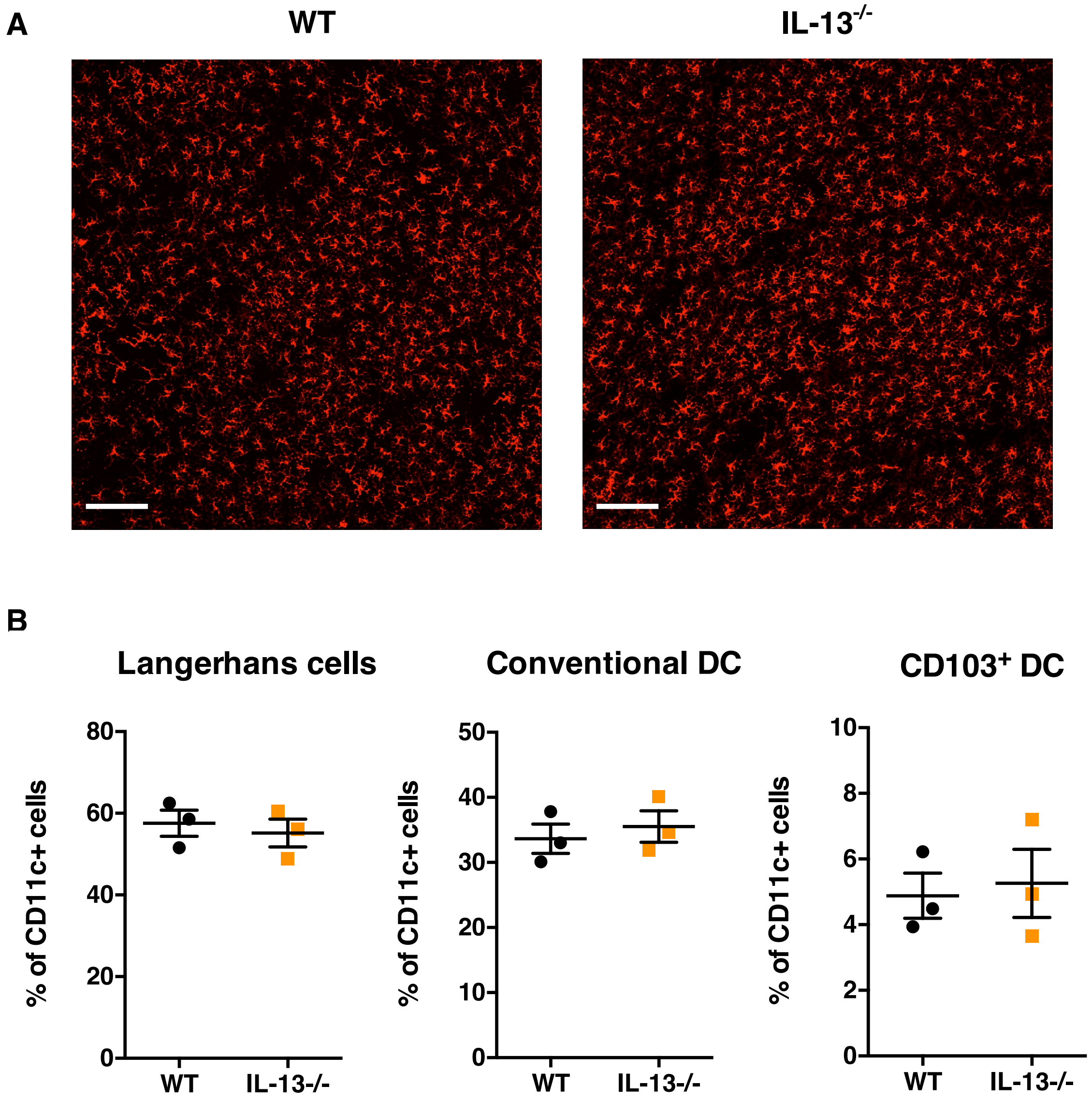

Supplementary Figure 8. Absence of IL-13 does not alter Langerhans cell networks or skin dendritic cell numbers

Naïve BALB/c WT and IL-13$^{-/-}$ mice were analysed for presence of Langerhans cells and dermal DC populations. (A) Epidermal sheets were collected and stained freshly for Langerin^{+} (red) cell networks. Epidermal sheets were analysed on a Leica Sp5 fluorescence microscope. 3 mice were analysed per group; a representative example of each group is shown. Original magnification x20. Scale bars = 100μm. (B) Whole skin was isolated and single cell suspensions were analysed by FACS for numbers of Langerhans cells (gated as CD45^{+}, CD64^{-}, CD11c^{+}, CD11b^{mid}, Langerin^{+}), conventional DC (gated as CD45^{+}, CD64^{-}, CD11c^{+}, CD11b^{hi}, Langerin^{-}) and CD103+ DC (gated as CD45^{+}, CD64^{-}, CD11c^{+}, CD11b^{lo}, Langerin^{+}, CD103^{+}, XCR1^{+}) (n=3 per group). Data is represented as % of total CD11c DC population.

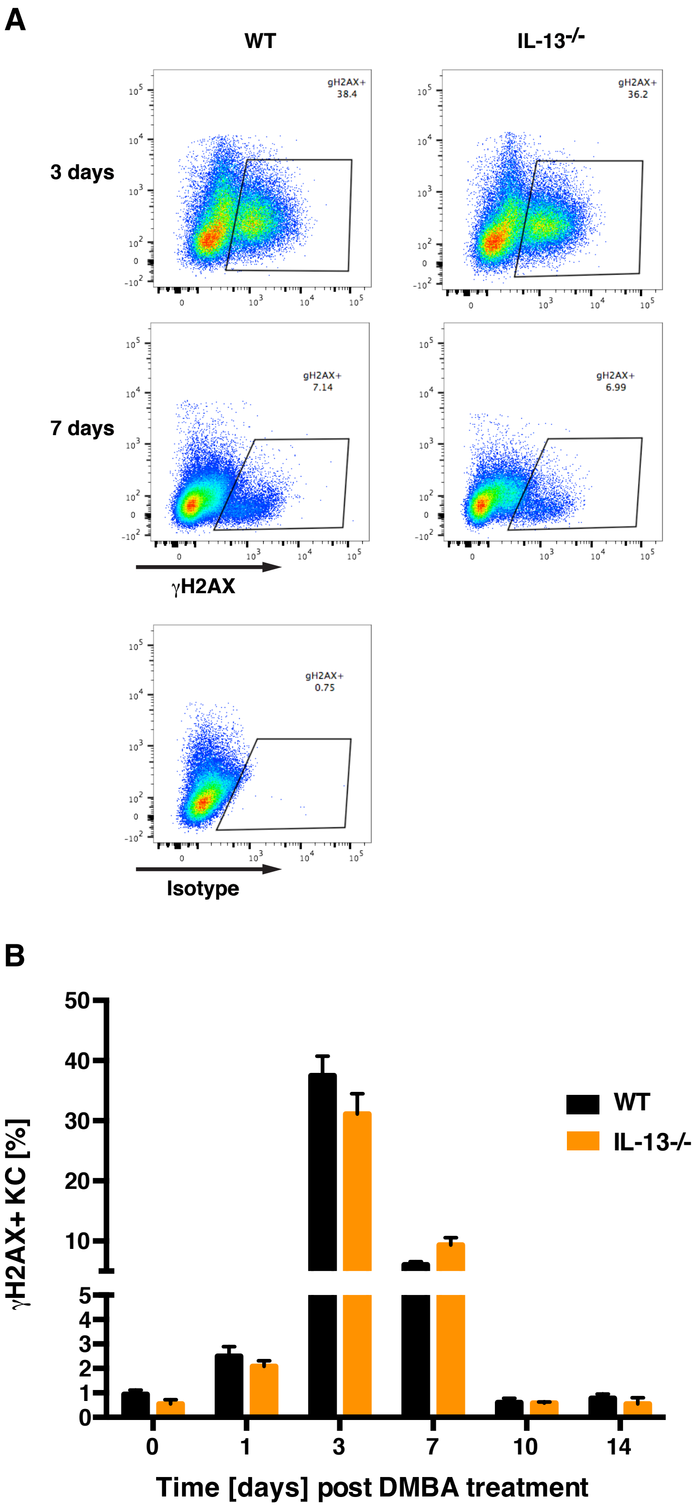

Supplementary Figure 9. Topical DMBA exposure induces similar level of EC DNA-damage in the absence of IL-13

BALB/c WT and IL-13$^{-/-}$ mice were treated with a single dose of 200nmol DMBA on the dorsal ear skin. At selected time-points thereafter epidermis was isolated and CD45^{-} KC stained for intranuclear γH2AX. γH2AX (the phosphorylation of the histone H2AX) flanks sites of double-stranded DNA breaks caused by the DMBA exposure. γH2AX is a sensitive marker for quantification of DNA-damage and repair processes. Dead cells were excluded from the analysis using a dead cell dye. (A) Representative staining of γH2AX at peak time-points with isotype control. (B) Quantification of % KC that have phosphorylated H2AX at varying time-points following DMBA in BALB/c WT (black bars) and IL-13$^{-/-}$ (orange bars) mice (n=3-14 mice per group per time-point). Data is represented as mean ± 1 SEM.

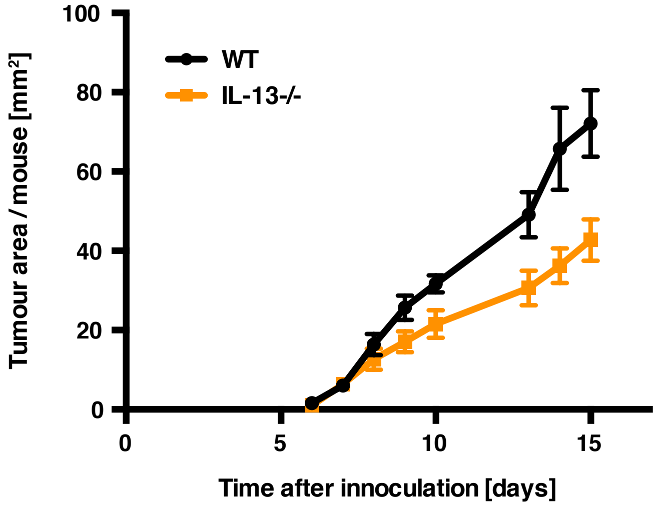

Supplementary Figure 10. The tumour protective effect of IL-13 is restricted to carcinogenesis at the epithelial skin barrier

BALB/c WT and IL-13$^{-/-}$ mice were inoculated subcutaneously in the flank with 10^4 CT26 cells and tumour growth measured over time. Tumour sizes were measured with calipers daily and the data shown as the average tumour area per mouse over time (n=7 per group).

A

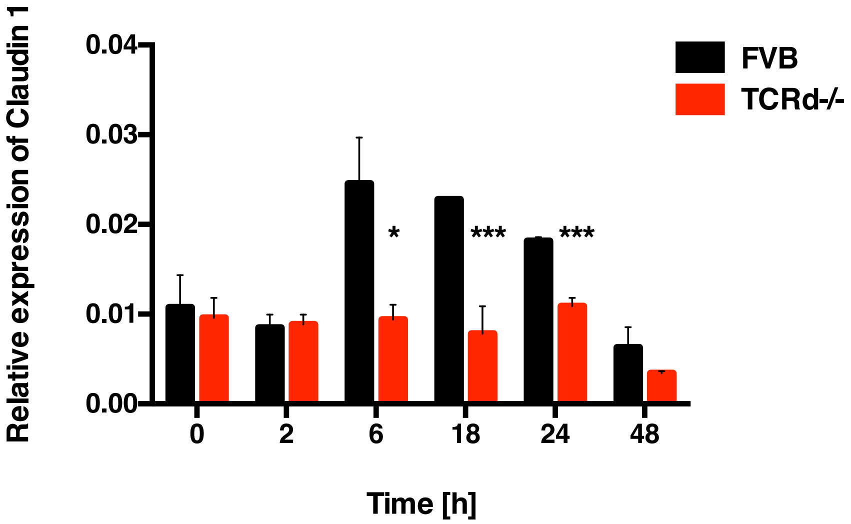

B

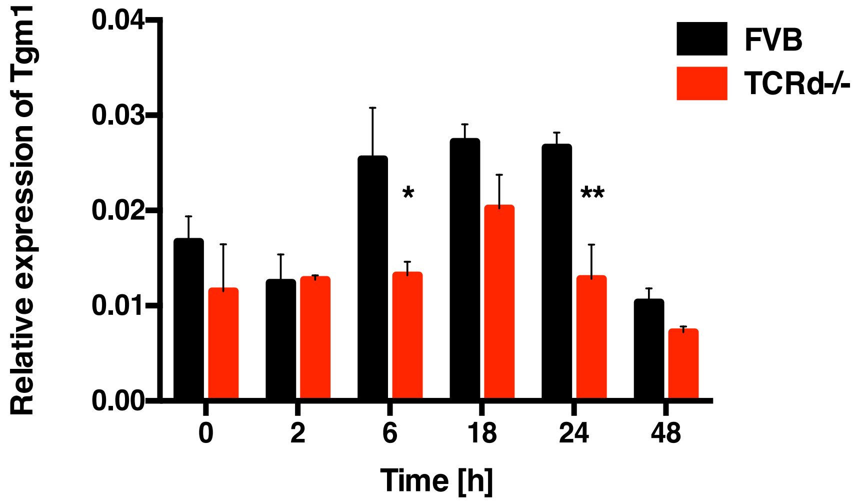

Supplementary Figure 11. IELs promote epithelial maturation following tape-stripping

Expression analysis of (A) claudin 1 and (B) transglutaminase 1 in isolated epidermis from FVB WT mice (black bars) and TCRd$^{-/-}$ mice (red bars) at indicated time-points after skin abrasion by tape-stripping (n=3 per time-point). Data was done by qRT-PCR and are expressed as mean ± 1 SEM relative to the control gene cyclophylin. Statistical significance of difference between experimental groups was determined using Student's t-test for unpaired data with * correlating to p<0.05, ** p<0.01 and *** p<0.001.

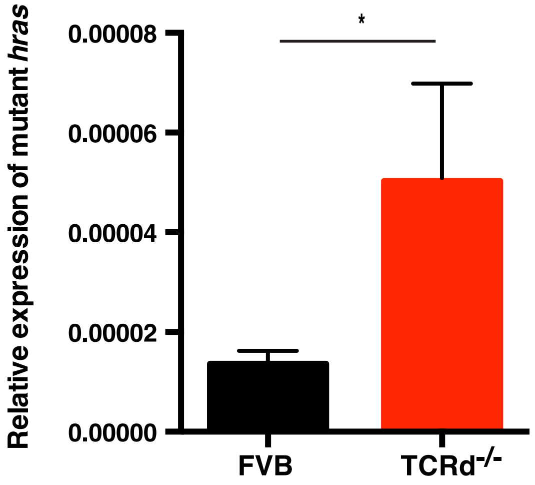

Supplementary Figure 12. In the absence of IELs *hras* mutations accumulate in the epidermis

FVB WT mice and TCRd$^{-/-}$ mice were treated with 200nmol DMBA on back skin once weekly for 6 weeks. Mice had no clinically apparent disease at this point. Epidermis was then collected and total genomic DNA extracted. A custom Taqman qRT-PCR assay for mutant *hras* (A->T transversion within *hras* codon 61) was performed and expression normalised against *Actb* (n=5 per group). Data is represented as mean ± 1 SEM. Statistical significance of difference between experimental groups was determined using Student's t-test for unpaired data with * correlating to $p<0.05$.
